# Health-related quality of life before and after management in adults referred to otolaryngology: rospective national study

**DOI:** 10.1111/j.1749-4486.2011.02433.x

**Published:** 2012-02

**Authors:** IRC Swan, FH Guy, MA Akeroyd

**Affiliations:** 1 MRC Institute of Hearing Research (Scottish Section), Glasgow Royal InfirmaryAlexandra Parade; 2 Department of Otolaryngology, University of GlasgowGlasgow, UK

## Abstract

**Objective:**

An assessment of the effect of otolaryngological management on the health-related quality of life of patients.

**Design:**

Application of the Health Utilities Index mark 3 (HUI-3) before and after treatment; application of the Glasgow Benefit Inventory (GBI) after treatment.

**Setting:**

Six otolaryngological departments around Scotland.

**Participants:**

A 9005 adult patients referred to outpatient clinics.

**Main outcome measures:**

Complete HUI-3 data was collected from 4422 patients; complete GBI data from 4235; complete HUI-3 and GBI data from 3884.

**Results:**

The overall change in health related quality of life from before to after management was just +0.02. In the majority of subgroups of data (classified by type of management) there was essentially no change in HUI-3 score. The major exceptions were those patients provided with a hearing aid (mean change 0.08) and those whose problem was managed surgically (mean change 0.04). The mean GBI score was 5.3 which is low. Those managed surgically reported a higher GBI score of 13.0.

**Conclusion:**

We found that patients treated surgically or given a hearing aid reported a significant improvement in their health related quality of life after treatment in otolaryngology departments. In general, patients treated in other ways reported no significant improvement. We argue that future research should look carefully at patient groups where there is unexpectedly little benefit from current treatment methods and consider more effective methods of management.

Traditionally, outcomes of medical care are based on clinical observations or laboratory measurements, but in recent years, there has been an increasing recognition that these need to be complemented by quantitative measures of the impact of the intervention on patients’ health status or health-related quality of life (‘HRQoL’). The three major measures are the Health Utilities Index mark 3 (HUI-3),^1^ the EQ-5D,^2^ and the SF-36^3^ questionnaires. These are generic questionnaires that are applicable to the full spectrum of health: their items cover experiences of illness, such as pain, fatigue, or disability, as well as broader aspects of the patient’s physical, emotional and social well-being. The importance of these measures is demonstrated by their wide use in the current National Services Scotland (NHS). For instance, the EQ-5D is the questionnaire used to assess the generic health status in the Patient Reported Outcome Measures (PROMs) for hip replacements, knee replacements, hernias and varicose veins.^4^ It is also recommended by the National Institute for Health and Clinical Excellence for the measurement and valuation of HRQoL in economic evaluations of health care.^5^

In audiology and otolaryngology, there have been studies of HRQoL in the specific domains of hearing aids^6–^^8^, cochlear implantation^9–^^12^ and head and neck cancer^13,^^14^. But there has been no large-scale HRQoL study of the general population of patients referred to otolaryngology clinics. We reasoned that such a study would be particularly useful as its outcomes would allow overall comparisons of otolaryngology with other specialities, as the majority of otolaryngology problems are neither life-threatening nor require surgery and so do not allow comparisons by mortality rates or surgical success rates. We therefore designed a suitable project to address this gap. We measured the baseline values of HRQoL of a broad spectrum of patients referred to six otolaryngology clinics across Scotland and then the changes in HRQoL owing to their otolaryngological management. We also collected relevant clinical data. The results are reported according to patients’ initial diagnosis and how they were managed.

## Methods

### Ethical considerations

The study was approved by the Scottish Multicentre Research Ethics Committee. Informed consent was obtained from all participants. All authors had full access to all the data.

### HRQoL questionnaires

We used two generic instruments, the HUI-3 and the Glasgow Benefit Inventory (‘GBI’). We chose the HUI-3 because it has been very widely used in health economic evaluations across all domains of health care, whereas we chose the GBI as it has been widely used in otolaryngology. The HUI-3 is a preference-based utility measure of generic health status, assessing patient preferences via 12 questions with four to six available responses for each. From these, an overall health utility measure is obtained via a weighted scoring algorithm, giving a single number ranging between 0 (death) and 1 (full health). The algorithm also gives scores on eight scales of health-status attributes, which also scale from 0 to 1: Vision, Hearing, Speech, Ambulation, Dexterity, Emotion, Cognition, and Pain. The HUI-3 is of relevance to otolaryngology (and indeed also to audiology) as it includes four questions on hearing and speech understanding (note that the EQ-5D does not include such questions and is mostly insensitive to the effects of impaired hearing or even from the ‘life-changing’ benefits gained from a cochlear implant^7,^^8,^^15,^^16^). To obtain measures of benefit from the HUI-3, we applied it both before and after management and then took the difference.

The GBI was designed to give an assessment of the patient’s perceived benefit from otolaryngological interventions^17,^^18^. It has 18 questions that ask directly about the change in health status resulting from management. The response to each question is based on a five-point Likert scale; these are then scaled and averaged to give a final score ranging between −100 and +100: negative scores represent a worse outcome, zero no change, and positive scores represent a benefit. As it was specifically designed as a benefit questionnaire, the GBI was applied once, after management.

### Procedure

**Fig. 1:**
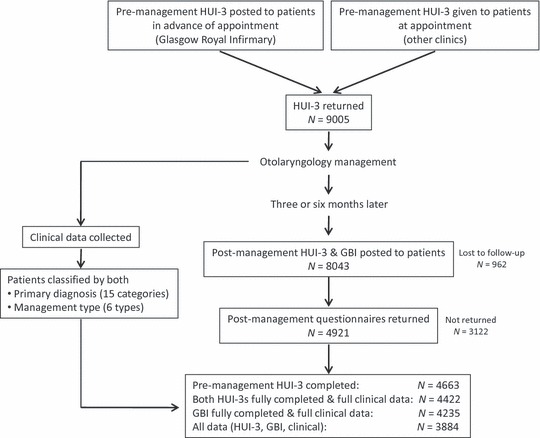
Overall design of the study.

Figure 1 shows a flowchart of the design. The study was run between 2001 and 2005, with post-management data collected up to May 2006, although the collection dates differed between departments. The aim was to include adult patients (14 years and older) attending any of six otolaryngology clinics in Scotland during the study period (see Table 1). Approximately 62 400 new patients were seen in these departments during the period of the study^19^, but the actual samples were affected by the workload and enthusiasm of the booking clerks and clinic receptionists and were considerably less than this. The selection was not influenced by the authors.

**Table 1:** Overall statistics of the study: reports the age and sex of those who fully completed both pre-and post-management HUI-3 questionnaires

	Royal Infirmary, Glasgow	Stobhill Hospital, Glasgow	Gartnavel Hospital, Glasgow	Crosshouse Hospital, Kilmarnock	Raigmore Hospital, Inverness	Royal Infirmary, Aberdeen	Total
Number	1575	213	203	305	134	1992	4422
Percentage of total	35.6	4.8	4.6	6.9	3.0	45.0	100
Males : Females (number)	650 : 925	93 : 120	80 : 123	125 : 180	55 : 79	892 : 1100	1895 : 2527
Males : Females (%)	41 : 59	44 : 56	39 : 61	41 : 59	41 : 59	45 : 55	43 : 57
Mean age (years)	56	54	54	57	55	52	54
Standard deviation of age (years)	16	17	14	16	16	17	16

HUI-3, Health Utilities Index mark 3.

The pre-management HUI-3 was either mailed to the patient 2 weeks before their appointment with a request to complete it and return it at their visit (Glasgow Royal Infirmary) or given to new patients on arrival for their first clinic appointment to be completed before consultation (other clinics); 9005 were returned (=14% of the maximum possible sample pool). Either three or 6 months after the completion of management, the post-management HUI-3 and the GBI were posted to patients. Three months was appropriate for those who did not require treatment or were treated medically as they commonly had more than one consultation, but as it would have been too short for recovery from a surgical procedure or for acclimatisation to hearing aids^20,^^21^, we waited 6 months in these groups. 8043 post-management questionnaires were sent out (the remaining 962 patients were lost to follow-up: either they defaulted from attendance, or their clinical data were not returned from the department they attended); 4921 were returned, giving a response rate of 61.2%. This return rate is comparable with other HRQoL studies where the subjects were asked by mail to complete the follow-up questionnaire.

Not every questionnaire was fully completed by each patient as the data were obtained by post, and therefore, we could not ensure that each question was answered. 4663 patients fully completed the pre-management HUI-3 questionnaire (94.8%), 4422 patients fully completed both the pre- and post-management HUI-3 questionnaires (89.8%), and 4235 patients fully completed the GBI (86.1%). The analyses below concentrate on the *N* = 4422 and *N* = 4235 groups. Their mean age was 54 years (standard deviation = 16 years), and the overall male–female split was 43−57%. About 80% of the patients were from either the Glasgow Royal Infirmary or the Aberdeen Royal Infirmary (see Table 1; for further detail, see Supplementary Table S1). Chi-square tests showed that there were no significant effects in the three contingency tables of the number of HUI-3 & GBI questionnaires returned by hospital (χ^2^ = 2.33, d.f. = 5, *P* = 0.80), by diagnosis category (χ^2^ = 2.48, d.f. = 14, *P* = 1.00), or by management type (χ^2^ = 0.87, d.f. = 5, *P* = 0.97).

The patients were classified into 15 diagnostic categories on the basis of copies of the letters sent by the clinicians to the patient’s family doctor after each visit to the clinic, which were read and coded by the lead author (see Table 2). The 15 categories fell into 4 broader classes of ear related, nose related, throat related, and other. The patients were also classified into six types of otolaryngological management (see Table 3).

**Table 2:** Main results: the change in Health Utilities Index mark (HUI) from pre-management to post-management and the GBI score after management. The data are grouped by diagnosis category. The other columns reported the number of people in each group and other information. Note that not everyone who completed both HUI-3s completed the (GBI), or vice versa, and so the numbers of people are not necessarily the same. The asterisked results were statistically significant after allowing for a 30-test Bonferroni correction

Region	Diagnosis Category	Number (HUI-3)	% (HUI-3)	Mean age, years (HUI-3)	Pre- management HUI-3 score	Change in HUI-3 score	Number (GBI)	% (GBI)	Mean age, years (GBI)	GBI score
Ear	Sensorineural hearing loss	947	21.3	60	0.566	0.044^*^	933	22.0	59	2.8^*^
Ear	Inactive middle ear disease	214	4.8	50	0.605	0.072^*^	215	5.1	49	8.6^*^
Ear	Active middle ear disease	158	3.6	55	0.478	0.084^*^	154	3.6	54	6.8^*^
Ear	External ear disease	208	4.7	53	0.620	0.056	205	4.8	52	8.9^*^
Ear	Dizziness	410	9.2	55	0.590	0.033	388	9.2	54	3.8^*^
Ear	Neurological problem	93	2.1	57	0.510	−0.026	84	2.0	55	−0.4
Nose	Nasal anatomical problem	341	7.8	49	0.723	−0.012	322	7.6	47	8.3^*^
Nose	Rhinosinusitis	573	13.0	51	0.728	0.008	543	12.8	50	6.5^*^
Nose	Snoring	65	1.5	47	0.758	−0.030	62	1.5	48	1.2
Throat	Throat Inflammation	204	4.6	37	0.752	0.038	205	4.8	36	12.0^*^
Throat	Benign Larynx	143	3.2	59	0.684	−0.017	141	3.3	59	7.2^*^
Throat	Benign Lump	148	3.3	51	0.744	0.004	137	3.2	50	7.3^*^
Throat	Gastro-oesophageal reflux/Globus	394	8.9	57	0.688	−0.029	353	8.3	55	3.7^*^
Other	Malignancy	34	0.8	66	0.686	−0.121	31	7.3	64	7.0
Other	No abnormality detected (NAD)	490	11.1	53	0.711	0.026	462	10.9	63	3.3
** **	Total	4422	100	54	0.650	0.021	4235	100	53	5.3

GBI, Glasgow Benefit Inventory; HUI-3, Health Utilities Index mark 3.

**Table 3:** As Table 2, but grouped by management type. The asterisked results were statistically significant after allowing for a 12-test Bonferroni correction

Classification	Definition	Number (HUI-3)	% (HUI-3)	Mean age, years (HUI-3)	Pre-management HUI-3 score	Change in HUI-3 score	Number (GBI)	% (GBI)	Mean age, year (GBI)	GBI score
‘Reassure’	= given reassurance or advice on self-management	1756	39.7	54	0.694	0.008	1700	40.0	53	1.7^*^
‘Medical treatment’	= topical or systemic medication	1055	23.9	55	0.665	0.004	978	23.0	53	5.2^*^
‘Therapy’	= speech therapy, vestibular exercises, or physiotherapy	222	5.0	64	0.584	0.017	511	12.1	63	6.6^*^
‘Hearing aid provision’	= hearing aid provided or replaced	534	12.1	58	0.452	0.084^*^	210	5.0	57	7.0^*^
‘Surgery’	= managed surgically	781	17.7	46	0.694	0.038^*^	762	18.0	45	13.0^*^
‘Refer on’	= referred to another specialist	74	1.7	52	0.580	−0.008	74	1.7	51	−1.8
	Total	4422	100	54	0.650	0.021	4235	100	53	5.3

GBI, Glasgow Benefit Inventory; HUI-3, Health Utilities Index mark 3.

### Comparison data

We know of no large-scale UK general-population data for comparing the HUI-3 scores to, so instead we used a Canadian data set (*n* = 61536) from the developers of the HUI-3.^22^ From the linear-regression coefficients reported, there we calculated the expected score for each of our participants given their sex and age (in decades), and then these values were used as comparison data. For the GBI, its definition as a benefit measure implies that the baseline value is zero.

### Statistical methods

To determine the statistical significance of our results, we used paired-sample Student t-tests (HUI-3), one-sample t-tests (GBI), Pearson correlations (HUI-3 *versus.* GBI), and chi-square tests (contingency tables), assuming an alpha of 0.05. Bonferonni corrections were applied to correct for multiple comparisons in each of the *t*-tests for change in HUI-3 score. We did not attempt to modify the sampling to equate the group sizes across all combinations of diagnosis and management type, even though some were relatively uncommon. Accordingly, in the text, we concentrate on those combinations with at least 20 people, chosen arbitrarily, although all the results are reported in the supplementary tables. All statistical calculations were carried out in PASW (SPSS) version 18 or Excel 2007.

## Results

### Health utilities index

Across the group of 4422 patients who fully completed both HUI-3 questionnaires, the mean pre-management HUI-3 score was 0.650 (SD = 0.307). The mean value for the comparison population was 0.83, so indicating that the overall HRQoL of the present sample was substantially poorer.

We found that the pre-management HUI-3 scores differed substantially across the 15 diagnostic categories, from 0.478 to 0.758 (see Table 2). There was a substantial variation in mean age across category, ranging from 37 years (throat inflammation) to 66 years (malignancy). There was a small overall correlation between age and pre-management HUI-3 score: the older the patient, the lower their reported score (*r* = −0.21, d.f. = 4621, *P* < 0.001), although it was much less than the corresponding correlation in the control group (*r* = −0.76, d.f. = 4921, *P* < 0.001). The pre-management HUI-3 scores also differed substantially across management type, from 0.452 for those provided with a hearing aid to 0.694 for both those reassured or managed surgically (see Supplementary Table S2).

The mean change in HUI-3 score from before to after (termed ‘Δ’) across all 4422 patients was +0.021 (standard deviation = 0.23). This was statistically significantly different from zero (*t* = 6.13, d.f. = 4421), although unsurprisingly so given the very large number of patients. The largest positive change in HUI-3 was found for the diagnosis category of active middle ear disease (Δ = 0.084); the largest negative change was for the malignancy category (Δ = −0.121). The change in HUI-3 was statistically significant for just three of the 15 diagnosis categories, all related to the ear: sensorineural hearing loss, active middle ear disease and inactive middle ear disease (see Table 2). It was also statistically significant for two management types (see Table 3), hearing aid provision and surgery (Δ = 0.084 and 0.038, respectively), but not for the four others. Of the 90 combinations of management type by diagnosis, only two gave a statistically significant change in HUI-3: hearing aid provision/sensorineural hearing loss (Δ = 0.08) and surgery/active middle ear disease (Δ = 0.16). Although some of the other combinations gave large changes, none were statistically significant after a Bonferroni correction for multiple comparisons was applied (see Supplementary Table S2). Table 4 summarises those combinations of management/diagnosis that gave a Δ of at least ±0.05^23^ and were based on at least 20 people. It is noteworthy that four of the five ear-related diagnosis categories appear at least once on the list for Δ > +0.05 – the exception is neurological problems, which instead is on the < −0.05 list – whereas none of the 11 diagnosis categories related to the nose, throat, or other appear on the > +0.05 list; though, three do on the < −0.05 list. It is perhaps to be expected that there was a substantial reduction in quality of life for surgery for malignancy of the head and neck (Δ = −0.159).

**4:** The combinations of management type/diagnosis category that gave the largest absolute changes in HUI-3 score, based on at least 20 people. The groups are sorted by the size of the change

Management type	Diagnosis category	Number	Pre-management HUI-3 score	Change in HUI-3 score	Gain or reduction in HUI-3 score?
Surgery	Active middle ear disease	62	0.478	+0.156	Gain
Surgery	Inactive middle ear disease	55	0.608	+0.139	Gain
Medical treatment	Dizziness	41	0.564	+0.099	Gain
Hearing aid provision	Inactive middle ear disease:	53	0.476	+0.085	Gain
Therapy	SNHL	42	0.494	+0.077	Gain
Hearing aid provision	SNHL	437	0.459	+0.084	Gain
Medical treatment	External ear disease:	143	0.630	+0.055	Gain
Reassure	External ear disease	45	0.680	+0.054	Gain
Reassure	Snoring	38	0.790	−0.051	Reduction
Medical treatment	Nasal anatomical problem	138	0.688	−0.054	Reduction
Refer on	Neurological problem	21	0.540	−0.055	Reduction
Surgery	Malignancy	26	0.724	−0.159	Reduction

HUI-3, Health Utilities Index mark 3.

Only one of the 48 combinations of HUI-3 *subscale* by management type gave a change as large as ±0.05: hearing subscale/hearing aid provision (Supplementary Table S3). Three combinations gave a statistically significant change: hearing subscale/hearing aid provision; speech subscale/hearing aid provision; and pain subscale/surgery. Each of these would be expected.

### Glasgow benefit inventory

4235 completed GBI questionnaires were returned. The mean score in the GBI was 5.32 (standard deviation = 17.3). This value was remarkably low given that the GBI scale is from −100 to +100, although it was statistically significantly different from 0 (*t* = 20.1, d.f. = 4234). When the values are divided by 100 to place them on the same scale as the change in HUI-3, which can range from −1 to +1, then the GBI score corresponds to 0.05, which is of the same order of magnitude as the overall changes in HUI-3 scores reported earlier.

When classified by diagnosis category (Table 2), the largest GBI scores were found for throat inflammation (12.0), external ear disease (8.9), and inactive middle ear disease (8.6; see Table 2). The scores for all but three diagnosis categories were significantly different from zero: the exceptions were neurological problems, snoring and malignancy. Of the six types of management (Table 3), surgery gave the highest mean GBI score (13.0). The other types gave GBI scores between 5 and 6 (hearing aid provision, medical treatment and therapy) or between −2 and + 2 (reassurance, refer on). All but refer on were significantly different from zero.

In contrast to the HUI-3 data, numerous combinations of diagnosis category and management type – including six of the 15 combinations with surgery – gave a significant effect after applying a Bonferroni correction for multiple comparisons (Supplementary Table S4).

### Health utilities index mark 3 *versus* Glasgow benefit inventory

3879 patients fully completed both HUI-3 questionnaires and the GBI questionnaire, representing 88% of those who completed both HUI-3s. The overall correlation between the change in HUI-3 and the GBI score was only +0.20, although given the very large sample size, this was statistically significant. When divided by management type, the largest correlation was for surgery (*r* = 0.270) and the lowest for reassurance (*r* = 0.164). When divided by diagnosis category, the correlations were between 0.1 and 0.3, excepting snoring (*r* = 0.478) and benign lump (*r* = 0.060). A comparison of the data in the Supplementary Tables indicates that the differences within diagnostic category by management type were more likely to be statistically significant with the GBI than with the change in HUI-3.

## Discussion

### Synopsis of key findings

This is the first major large-scale study of the health-related quality of life (HRQoL) of patients referred to otolaryngology clinics. Over 4400 patients completed both HUI-3 questionnaires. We believe the general distribution of symptoms and diagnoses to be reasonably typical of otolaryngological referrals across the UK.

With some notable exceptions discussed later, the overall change in HRQoL owing to otolaryngological management was disappointingly small. The mean increase in HUI-3 score from pre-management to post-management was only 0.021, on a scale from 0 to 1; the benefit measured using the GBI was only 5.3, on a scale from −100 to + 100.

We classified the otolaryngological intervention into 6 overall types. Both surgical management and hearing aid provision groups gave statistically significant increases in HRQoL of, respectively, 0.084 and 0.038, whereas the other four types gave insignificant changes of therapy (0.017), reassurance (0.008), medical treatment (0.004), and refer on (−0.008). The size of the effects in some of the groups is broadly comparable with that observed in prior studies: for instance, we observed a mean change of 0.084 for the 437 patients with SNHL treated by a hearing aid, whereas Grutters *et al.*^7^ reported a mean change of 0.12 in the HUI-3 in patients after hearing aid fitting.

These results are all encouraging and clearly demonstrate that some – although only some –otolaryngological interventions can lead to measurable increases in quality of life when measured using a generic instrument.

### Does one always expect an improvement in HRQoL?

When the patients were subdivided by diagnosis category, the results showed that only some diagnoses and management types gave increases in HRQoL. We found that there was a statistically significant increase in HRQoL for just two subdivisions: sensorineural hearing loss treated by hearing aid provision and those with active middle ear disease treated surgically. Although part of this effect may be a small sample size in some subdivisions (further discussed later), it is clear that many combinations of diagnosis and management type led to only minimal effects on HRQoL.

A difference of 0.05 is often taken as a meaningful change in HUI-3 score.^23^. For only the surgery and hearing aid groups were there substantially more patients – around 20%– with a change >0.05 than with < −0.05 (Table S5). Across all 4422 patients, there were only 8% more patients with a change >0.05 than with < −0.05. It is clear that only a fraction of the patients seen in otolaryngology clinics report a benefit in HRQoL.

Undoubtedly, there are many patients referred to otolaryngology where no improvement in HRQoL is to be expected, as they are referred for a diagnosis rather than the expectation of successful management. One example is patients with a feeling of a lump in the throat. Their referral is principally to exclude malignancy, and these patients are unlikely to have any detrimental impact on their HRQoL from their condition, other than concern about possible diagnoses, and the treatment is unlikely to change it. Our results bore out this expectation: the mean increase in HUI-3 score for those whose primary symptom was benign lump was 0.004 (*N* = 148). We argue that these results indicate the limitations of a generic instrument: that the corresponding GBI score was 7.6 (*N* = 126) indicates that there are aspects to health that are not captured by the HUI-3. There are other groups of patients for whom management has been shown to be effective by use of disease-specific questionnaires, for example the SNOT questionnaire applied to rhinosinusitis^24^. In our sample, this particular group reported no significant improvement in either HUI-3 (0.008) or the GBI score (6.5). Disease-specific questionnaires are more likely to detect changes that occur in that particular condition, but they do not allow comparison with treatment for other conditions.

For many other conditions, there is no effective cure, for example tinnitus or mild unilateral hearing impairment, and here, the aim of otolaryngological management is to offer reassurance and advice. In other cases, the management is to encourage patients to make changes to their lifestyle: for example for those with laryngitis to stop smoking or those who snore to lose weight. These groups are unlikely to think that their HRQoL has improved in the short term, although it is hoped that, in some at least, their health will improve in the long term if they make the recommended lifestyle changes. It is of interest that for those diagnosed with dizziness, the mean change in HRQoL was small (0.033), but the range of scores was wide: some patients noticed a large improvement in HRQoL, while others felt that their situation had become worse. This is a large patient group with a poor HRQoL who are often not well managed. We note that the problem has been recognised nationally, and the efforts are being made to improve management.

### Strengths and weaknesses

The primary strength of this study is its large-scale, comprehensive design, deliberately covering all aspects of ENT and including patients from across Scotland. The results quantify HRQoL across the whole discipline of ENT, and the concomitant proportions of diagnoses seen and otolaryngological managements used may encourage future clinical audits.

There are some potential weaknesses, however. First, although over 4000 people completed the questionnaires, around 62000 people attended all six participating ENT clinics during the period of the project. We therefore only sampled a small proportion of the eligible population. The initial distribution of questionnaires was made by the clinics’ booking clerks and clinic receptionists and undoubtedly fluctuated across clinic and time. We did not influence their work in anyway. We therefore argue that the sample is effectively random, and regard it as unlikely that there was any substantial systematic bias for or against any group of patients which could have affected the results (although we note that malignancy may be under-represented, as such patients are often seen urgently and thus would have bypassed the project, and their treatment is often prolonged such that they would not have been asked to complete post-management questionnaires). A corollary is that we could not vary the sampling to ensure that all groups would be equally sized, as some diagnoses were somewhat rare; for instance, only 65 participants had a diagnosis of snoring. The result is a reduction in statistical power of some comparisons, but we felt that this was outweighed by the pragmatic disadvantage of continuing sampling, perhaps for many years, until all groups were equally sized. We also note that our classification of management types was, by necessity, somewhat broad, and for simplicity, we stipulated that each particular intervention could only fall into one overall type. In some circumstances, this could be questioned: for instance, one can argue that *surgery* for the diagnosis of lumps could also be classified as *reassurance*, as in most cases, the clinical result is that the lump was indeed benign. The full data presented in the Supplementary Tables will help if such details are of interest, and we hope that the results will suggest targeted follow-up work looking at specific categories of diagnosis or management.

Second, about 30% of those who were sent the post-management questionnaires did not return them. There were no substantial variations in this across the six clinics (Table 1). Analysis showed that there was a similar distribution of patients across clinic and diagnostic category between those who did not return the questionnaires and those who did. There was only a minimal difference in the mean pre-management HUI-3 score (0.66 *versus.* 0.65), but the age of those who returned the questionnaires was substantially older (54 *versus.* 46 years). It is not obvious why the return rate was higher in older patients; perhaps, they had more time available to complete the questionnaires.

Third, we found that the overall HRQoL of the patients was poorer in comparison with an age- and sex-matched normal Canadian population^22^, the values being 0.65 *versus.* 0.83. This is a surprisingly large difference on which future studies may be warranted. It may be related to the likelihood that many otolaryngological patients often have other unrelated medical problems and are on several different medications unrelated to their otolaryngological condition. Many conditions common in the elderly can have a large effect on HRQoL: the cited Canadian data showed an overall reduction of 0.16 from reference for patients with arthritis and 0.39 for those who had a stroke. But there may also be an overall factor of general health, as it is known that the general health of the population in Scotland is poor in comparison with other countries and, indeed, varies considerably within Scotland: we note that the mean HRQoL for the patients attending the Glasgow Royal Infirmary was only 0.57, whereas the values for the other hospital were 0.63 (Crosshouse), 0.67 (Stobhill) and 0.70 (Gartnavel, Raigmore and Aberdeen Royal Infirmary).

## Conclusion

We measured the health-related quality of life using the HUI-3 of 4422 patients referred to six otolaryngology clinics across Scotland. Patients were classified according to their diagnosis and how they were managed. We found that some groups gave a statistically significant improvement in their health-related quality of life after treatment in otolaryngology departments but many did not. 4235 patients also completed the GBI questionnaire. Its results were loosely related to the HUI-3 results, although there were more groups that gave a significant benefit. The largest changes in HUI-3 were seen in patients provided with a hearing aid (mean change 0.084) and those managed surgically (mean change, 0.038); the largest GBI scores were seen in those managed surgically (13.2), by therapy (7.0), and provided with a hearing aid (6.6.). We argue that future research should look carefully at patient groups where there is little benefit from current treatment methods and consider more effective methods of management.

**Keypoints**

- Only a small proportion of patients referred to otolaryngology in Scotland have a significant improvement in their health-related quality of life (HUI-3).
- Those patients provided with a hearing aid or managed surgically reported a statistically significant overall improvement in their health-related quality of life (HUI-3).

## Supporting Information

**Table S1**. The number of questionnaires returnedat each stage of the study for each of the hospitalsparticipating.

**Table S2.** Values of pre-management HUI-3 scoreand the pre-to-post change in HUI-3 score for the 4422 people whofully completed both pre- and post-management HUI-3 questionnairesfor every combination of diagnostic category (rows) and managementtype (columns). The asterisked differences were statisticallysignificant after allowing for a 36-test Bonferroni correction.‘Δ’ refers to the mean change in HUI-3 from before to after.

**Table S3**. Values of the pre-to-post change inHUI-3 score for each subscale of the HUI-3. The data is for the4422 people who fully completed both pre- and post-management HUI-3questionnaires. The asterisked results were statisticallysignificant after allowing for a 27-test Bonferroni correction.

**Table S4**. As Table S1 but for the values of thepost-management GBI. The data is for the 4235 people who fullycompleted it.

**Table S5.** The numbers of patients whose changein HUI-3 score was larger than 0.05, <−0.05, orin-between, expressed as proportions. The management typesare sorted in order of the positive *vs.* negative difference.The ‘random data’ row reports what would be expectedgiven no change at all. It was calculated using a numericalsimulation of normally-distributed data with a standard deviationof 0.3 (about that of the our data) and with a test-retestcorrelation of 0.75 (about that of the HUI-3; e.g., Jones et al2005^24^).
