# Supplementary material for: Health-related quality of life before and after management in adults referred to otolaryngology: rospective national study
Source: Clin Otolaryngol. 2012 Feb;37(1):35–43. doi: 10.1111/j.1749-4486.2011.02433.x (PMC3380566; doi:10.1111/j.1749-4486.2011.02433.x)
Supplement: Supplementary file 1 [file coa0037-0035-SD1.doc]

**Patient reported outcome measures in otolaryngology**

**Swan, IRC, Guy, F, Akeroyd, MA**

**SUPPLEMENTARY TABLES**

**Table 1: The number of questionnaires returned at each stage of the study**

|  | **Royal**  **Infirmary,**  **Glasgow** | **Stobhill**  **Hospital, Glasgow** | **Gartnavel**  **Hospital,**  **Glasgow** | **Crosshouse**  **Hospital,**  **Kilmarnock** | **Raigmore**  **Hospital,**  **Inverness** | **Royal**  **Infirmary,**  **Aberdeen** | **Total** |
| --- | --- | --- | --- | --- | --- | --- | --- |
|  |  |  |  |  |  |  |  |
| **Number of post-management**  **questionnaires sent out** | 2878 | 456 | 449 | 502 | 247 | 3511 | **8043** |
| **Number of post-management**  **questionnaires returned** | 1747 | 246 | 229 | 333 | 156 | 2210 | **4921** |
| **Percentage of post-management**  **questionnaires returned** | 61% | 54% | 51% | 66% | 63% | 63% | **61%** |
| **Number of fully-complete**  **pre- & post- HUI3** | 1575 | 213 | 203 | 305 | 134 | 1992 | **4422** |
| **Number of fully-complete GBI** | 1449 | 213 | 196 | 287 | 126 | 1964 | **4235** |
| **Number of fully-complete**  **pre- & post- HUI-3 & GBI** | 1338 | 187 | 180 | 262 | 115 | 1797 | **3879** |

**Table S2: Values of pre-management HUI-3 score and the pre-to-post change in HUI-3 score for the 4422 people who fully completed both pre- and post-management HUI-3 questionnaires. The data is organized by diagnostic category (rows) and management type (columns). The two asterisked differences were statistically significant after allowing for a 90-test Bonferroni correction. “Δ” refers to the mean change in HUI-3 from before to after.**

| **Region** | **Diagnosis Category** |  |  | **Reassure** |  |  |  | **Medical treatment** | |  |  | **Therapy** |  |  |
| --- | --- | --- | --- | --- | --- | --- | --- | --- | --- | --- | --- | --- | --- | --- |
|  |  |  | **N** | **Pre-mgmt HUI-3** | **Δ** |  | **N** | **Pre-mgmt HUI-3** | **Δ** |  | **N** | **Pre-mgmt HUI-3** | **Δ** |  |
|  |  |  |  |  |  |  |  |  |  |  |  |  |  |  |
|  |  |  |  |  |  |  |  |  |  |  |  |  |  |  |
| Ear | **Sensorineural hearing loss** |  | 438 | 0.682 | **0.006** |  | 20 | 0.459 | **0.003** |  | 42 | 0.494 | **0.077** |  |
| Ear | **Inactive middle-ear disease** |  | 92 | 0.662 | **0.026** |  | 13 | 0.731 | **0.041** |  | 1 | 0.468 | **0.477** |  |
| Ear | **Active middle-ear disease** |  | 15 | 0.682 | **-0.099** |  | 62 | 0.473 | **0.042** |  | 1 | 0.599 | **-0.117** |  |
| Ear | **External ear disease** |  | 45 | 0.680 | **0.054** |  | 143 | 0.630 | **0.055** |  | 0 |  |  |  |
| Ear | **Dizziness** |  | 276 | 0.633 | **0.016** |  | 41 | 0.564 | **0.099** |  | 67 | 0.519 | **0.018** |  |
| Ear | **Neurological problem** |  | 45 | 0.550 | **-0.017** |  | 9 | 0.547 | **-0.006** |  | 12 | 0.349 | **-0.058** |  |
|  |  |  |  |  |  |  |  |  |  |  |  |  |  |  |
| Nose | **Nasal anatomical problem** |  | 48 | 0.712 | **0.027** |  | 138 | 0.688 | **-0.054** |  | 0 |  |  |  |
| Nose | **Rhinosinusitis** |  | 36 | 0.700 | **-0.030** |  | 366 | 0.717 | **0.000** |  | 0 |  |  |  |
| Nose | **Snoring** |  | 38 | 0.790 | **-0.051** |  | 3 | 0.494 | **0.193** |  | 0 |  |  |  |
|  |  |  |  |  |  |  |  |  |  |  |  |  |  |  |
| Throat | **Throat Inflammation** |  | 72 | 0.768 | **0.022** |  | 7 | 0.800 | **0.010** |  | 2 | 0.703 | **0.270** |  |
| Throat | **Benign Larynx** |  | 44 | 0.634 | **-0.029** |  | 7 | 0.768 | **-0.082** |  | 44 | 0.712 | **-0.034** |  |
| Throat | **Benign Lump** |  | 91 | 0.773 | **0.003** |  | 6 | 0.598 | **-0.001** |  | 1 | 0.685 | **0.234** |  |
| Throat | **Gastro-oesophageal reflux (GOR) / Globus** |  | 134 | 0.759 | **-0.030** |  | 198 | 0.671 | **-0.025** |  | 3 | 0.347 | **-0.105** |  |
|  |  |  |  |  |  |  |  |  |  |  |  |  |  |  |
| Other | **Malignancy** |  | 4 | 0.621 | **0.021** |  | 0 |  |  |  | 0 |  |  |  |
| Other | **No abnormality detected (NAD)** |  | 378 | 0.719 | **0.023** |  | 42 | 0.699 | **0.039** |  | 49 | 0.701 | **0.015** |  |
|  |  |  |  |  |  |  |  |  |  |  |  |  |  |  |
|  | **Total / Means** |  | **1756** | **0.694** | **0.008** |  | **1055** | **0.665** | **0.004** |  | **222** | **0.584** | **0.017** |  |

| **Region** | **Diagnosis Category** |  |  | **Hearing-aid provision** |  |  |  | **Surgical treatment** | |  |  | **Refer on** |  |  |
| --- | --- | --- | --- | --- | --- | --- | --- | --- | --- | --- | --- | --- | --- | --- |
|  |  |  | **N** | **Pre-mgmt HUI-3** | **Δ** |  | **N** | **Pre-mgmt HUI-3** | **Δ** |  | **N** | **Pre-mgmt HUI-3** | **Δ** |  |
|  |  |  |  |  |  |  |  |  |  |  |  |  |  |  |
|  |  |  |  |  |  |  |  |  |  |  |  |  |  |  |
| Ear | **Sensorineural hearing loss** |  | 437 | 0.459 | **0.084 *** |  | 7 | 0.581 | **0.038** |  | 3 | 0.787 | **-0.389** |  |
| Ear | **Inactive middle-ear disease** |  | 53 | 0.476 | **0.085** |  | 55 | 0.608 | **0.139** |  | 0 | 0. |  |  |
| Ear | **Active middle-ear disease** |  | 17 | 0.322 | **0.143** |  | 62 | 0.478 | **0.156 *** |  | 1 | 0.285 | **0.214** |  |
| Ear | **External ear disease** |  | 10 | 0.386 | **0.061** |  | 10 | 0.433 | **0.084** |  | 0 | 0. |  |  |
| Ear | **Dizziness** |  | 14 | 0.411 | **0.034** |  | 7 | 0.261 | **0.304** |  | 5 | 0.321 | **0.269** |  |
| Ear | **Neurological problem** |  | 2 | 0.443 | **0.043** |  | 4 | 0.339 | **0.038** |  | 21 | 0.540 | **-0.055** |  |
|  |  |  |  |  |  |  |  |  |  |  |  |  |  |  |
| Nose | **Nasal anatomical problem** |  | 0 |  |  |  | 152 | 0.756 | **0.015** |  | 3 | 0.792 | **-0.100** |  |
| Nose | **Rhinosinusitis** |  | 0 |  |  |  | 170 | 0.759 | **0.035** |  | 1 | 0.778 | **0.023** |  |
| Nose | **Snoring** |  | 0 |  |  |  | 15 | 0.810 | **-0.035** |  | 9 | 0.624 | **-0.008** |  |
|  |  |  |  |  |  |  |  |  |  |  |  |  |  |  |
| Throat | **Throat Inflammation** |  | 0 |  |  |  | 111 | 0.763 | **0.049** |  | 12 | 0.540 | **0.011** |  |
| Throat | **Benign Larynx** |  | 0 |  |  |  | 47 | 0.700 | **0.008** |  | 1 | 0.364 | **0.475** |  |
| Throat | **Benign Lump** |  | 0 |  |  |  | 44 | 0.695 | **-0.004** |  | 6 | 0.813 | **0.060** |  |
| Throat | **Gastro-oesophageal reflux (GOR) / Globus** |  | 1 | -0.280 | **0.056** |  | 55 | 0.617 | **-0.032** |  | 3 | 0.613 | **-0.121** |  |
|  |  |  |  |  |  |  |  |  |  |  |  |  |  |  |
| Other | **Malignancy** |  | 0 |  |  |  | 26 | 0.724 | **-0.159** |  | 4 | 0.504 | **-0.015** |  |
| Other | **No abnormality detected (NAD)** |  | 0 |  |  |  | 16 | 0.622 | **0.088** |  | 5 | 0.598 | **-0.010** |  |
|  |  |  |  |  |  |  |  |  |  |  |  |  |  |  |
|  | **Total / Means** |  | **534** | **0.452** | **0.084** |  | **781** | **0.694** | **0.038** |  | **74** | **0.580** | **-0.008** |  |

**Table S3: Values of the pre-to-post change in HUI-3 score for each subscale of the HUI-3. The data is for the 4422 people who fully completed both pre- and post-management HUI-3 questionnaires and is organized by management type (columns). The asterisked results were statistically significant after allowing for a 27-test Bonferroni correction**

| **HUI-3 Subscale** |  | **Reassure** |  | **Medical treatment** |  | **Therapy** |  | **Hearing-aid provision** |  | **Surgical treatment** |  | **Refer on** |  | **Overall** |
| --- | --- | --- | --- | --- | --- | --- | --- | --- | --- | --- | --- | --- | --- | --- |
|  |  |  |  |  |  |  |  |  |  |  |  |  |  |  |
| **Number** |  | **1756** |  | **1055** |  | **222** |  | **534** |  | **781** |  | **74** |  | **4422** |
|  |  |  |  |  |  |  |  |  |  |  |  |  |  |  |
|  |  |  |  |  |  |  |  |  |  |  |  |  |  |  |
| **Vision subscale** |  | -0.004 |  | -0.001 |  | +0.016 |  | +0.002 |  | -0.002 |  | +0.000 |  | **-0.001** |
| **Hearing subscale** |  | +0.012 |  | +0.022 |  | -0.025 |  | +0.191 * |  | +0.030 |  | +0.004 |  | **+0.037** |
| **Speech subscale** |  | +0.006 |  | +0.003 |  | +0.001 |  | +0.023 * |  | +0.003 |  | +0.020 |  | **+0.007** |
| **Ambulation subscale** |  | -0.002 |  | -0.008 |  | -0.010 |  | -0.007 |  | -0.001 |  | +0.011 |  | **-0.004** |
| **Dexterity subscale** |  | -0.006 |  | -0.004 |  | +0.001 |  | -0.005 |  | -0.003 |  | +0.014 |  | **-0.004** |
| **Emotion subscale** |  | -0.002 |  | +0.001 |  | +0.006 |  | +0.004 |  | +0.005 |  | -0.008 |  | **+0.001** |
| **Cognition subscale** |  | -0.004 |  | -0.012 |  | -0.013 |  | +0.004 |  | -0.013 |  | -0.025 |  | **-0.007** |
| **Pain subscale** |  | +0.006 |  | -0.001 |  | +0.016 |  | -0.008 |  | +0.043 * |  | -0.036 |  | **+0.009** |

**Table S4: As Table S1 but for the values of the post-management GBI. The data is for the 4235 people who fully completed it. The asterisked results were statistically significant after allowing for a 67-test Bonferroni correction**

| **Region** | **Diagnosis Category** |  |  |  |  | |  |  |  |  |  |  |  |  |
| --- | --- | --- | --- | --- | --- | --- | --- | --- | --- | --- | --- | --- | --- | --- |
|  |  |  | **Reassure** | |  |  | | **Medical treatment** | |  |  | **Therapy** | |  |
|  |  |  | **Number** | **GBI** |  |  | | **Number** | **GBI** |  |  | **Number** | **GBI** |  |
|  |  |  |  |  |  |  | |  |  |  |  |  |  |  |
| Ear | Sensorineural hearing loss |  | 447 | -0.63 |  |  | | 15 | 0.37 |  |  | 40 | 1.32 |  |
| Ear | Inactive middle-ear disease |  | 90 | 1.91 |  |  | | 13 | 10.26 |  |  | 1 | 11.11 |  |
| Ear | Active middle-ear disease |  | 17 | 0.00 |  |  | | 59 | 4.76 |  |  | 1 | 0.00 |  |
| Ear | External ear disease |  | 42 | 4.50 |  |  | | 147 | 11.02 * |  |  | 0 |  |  |
| Ear | Dizziness |  | 262 | 2.27 |  |  | | 38 | 5.48 |  |  | 62 | 9.99 |  |
| Ear | Neurological problem |  | 43 | -1.16 |  |  | | 9 | 11.73 |  |  | 7 | -2.78 |  |
|  |  |  |  |  |  |  | |  |  |  |  |  |  |  |
| Nose | Nasal anatomical problem |  | 48 | 1.91 |  |  | | 123 | 5.10 |  |  | 0 |  |  |
| Nose | Rhinosinusitis |  | 35 | -3.89 |  |  | | 342 | 3.33 * |  |  | 0 |  |  |
| Nose | Snoring |  | 36 | 1.23 |  |  | | 4 | 9.72 |  |  | 0 |  |  |
|  |  |  |  |  |  |  | |  |  |  |  |  |  |  |
| Throat | Throat Inflammation |  | 72 | 2.70 |  |  | | 6 | -1.39 |  |  | 2 | 9.72 |  |
| Throat | Benign Larynx |  | 42 | 3.04 |  |  | | 6 | 8.33 |  |  | 43 | 6.52 |  |
| Throat | Benign Lump |  | 81 | 7.48 |  |  | | 6 | 9.72 |  |  | 1 | 2.78 |  |
| Throat | Gastro-oesophageal reflux (GOR) / Globus |  | 127 | 2.87 |  |  | | 170 | 4.66 * |  |  | 5 | -4.44 |  |
|  |  |  |  |  |  |  | |  |  |  |  |  |  |  |
| Other | Malignancy |  | 3 | 20.37 |  |  | | 0 |  |  |  | 0 |  |  |
| Other | No abnormality detected (NAD) |  | 355 | 2.39 * |  |  | | 40 | .90 |  |  | 48 | 10.94 |  |
|  |  |  |  |  |  |  | |  |  |  |  |  |  |  |
|  | **Total / means** |  | **1700** | **1.66** |  |  | | **978** | **5.20** |  |  | **210** | **7.00** |  |

| **Region** | **Diagnosis Category** |  |  |  |  | |  |  |  |  |  |  |  |  |
| --- | --- | --- | --- | --- | --- | --- | --- | --- | --- | --- | --- | --- | --- | --- |
|  |  |  | **Hearing-aid provision** | |  |  | | **Surgical treatment** | |  |  | **Refer on** | |  |
|  |  |  | **Number** | **GBI** |  |  | | **Number** | **GBI** |  |  | **Number** | **GBI** |  |
|  |  |  |  |  |  |  | |  |  |  |  |  |  |  |
| Ear | Sensorineural hearing loss |  | 421 | 6.71 * |  |  | | 7 | 0.00 |  |  | 3 | -1.85 |  |
| Ear | Inactive middle-ear disease |  | 53 | 7.86 |  |  | | 58 | 19.40 * |  |  | 0 |  |  |
| Ear | Active middle-ear disease |  | 13 | 10.26 |  |  | | 63 | 10.10 * |  |  | 1 | -2.78 |  |
| Ear | External ear disease |  | 6 | 9.26 |  |  | | 10 | -3.61 |  |  | 0 | . |  |
| Ear | Dizziness |  | 16 | -1.22 |  |  | | 7 | 12.70 |  |  | 3 | -8.33 |  |
| Ear | Neurological problem |  | 1 | 0.00 |  |  | | 3 | 5.56 |  |  | 21 | -4.10 |  |
|  |  |  |  |  |  |  | |  |  |  |  |  |  |  |
| Nose | Nasal anatomical problem |  | 0 |  |  |  | | 148 | 13.21 * |  |  | 3 | .00 |  |
| Nose | Rhinosinusitis |  | 0 |  |  |  | | 165 | 15.39 * |  |  | 1 | .00 |  |
| Nose | Snoring |  | 0 |  |  |  | | 14 | 2.18 |  |  | 8 | -4.86 |  |
|  |  |  |  |  |  |  | |  |  |  |  |  |  |  |
| Throat | Throat Inflammation |  | 0 |  |  |  | | 111 | 19.87 * |  |  | 14 | 2.78 |  |
| Throat | Benign Larynx |  | 0 |  |  |  | | 49 | 11.34 * |  |  | 1 | 2.78 |  |
| Throat | Benign Lump |  | 0 |  |  |  | | 43 | 7.69 |  |  | 6 | 0.00 |  |
| Throat | Gastro-oesophageal reflux (GOR) / Globus |  | 1 | -52.78 |  |  | | 47 | 5.91 |  |  | 3 | -23.15 |  |
|  |  |  |  |  |  |  | |  |  |  |  |  |  |  |
| Other | Malignancy |  | 0 |  |  |  | | 23 | 5.19 |  |  | 5 | 7.22 |  |
| Other | No abnormality detected (NAD) |  | 0 |  |  |  | | 14 | 6.55 |  |  | 5 | 3.89 |  |
|  |  |  |  |  |  |  | |  |  |  |  |  |  |  |
|  | **Total / means** |  | **511** | **6.57** |  |  | | **762** | **13.04** |  |  | **74** | **-1.76** |  |

**Table S5: The numbers of patients whose change in HUI-3 score was larger than 0.05, less than -0.05, or in-between, expressed as proportions. The management types are sorted in order of the positive *vs.* negative difference. The “random data” row reports what would be expected given no change at all. It was calculated using a numerical simulation of normally-distributed data with a standard deviation of 0.3 (about that of the our data) and with a test-retest correlation of 0.75 (about that of the HUI-3; e.g., Jones et al 2005 ^24^)**

| **Management Type** | **N** | **Change in HUI-3 < -0.05** | **Positive-to-negative difference** | **Change in HUI-3> 0.05** | **Imbalance**  **= P - Z** |
| --- | --- | --- | --- | --- | --- |
|  |  |  |  |  |  |
| **Hearing-aid** | 534 | 31% | 14% | 54% | 24% |
| **Surgery** | 781 | 29% | 24% | 47% | 18% |
| **Therapy** | 222 | 37% | 19% | 44% | 7% |
| **Reassure** | 1756 | 33% | 30% | 38% | 4% |
| **Clinical** | 1055 | 35% | 28% | 37% | 2% |
| **Refer-on** | 74 | 42% | 18% | 41% | 1% |
|  |  |  |  |  |  |
| **Overall** | **4422** | **33%** | **26%** | **41%** | **8%** |
|  |  |  |  |  |  |
| **Random data** | --- | 41% | 19% | 41% | 0% |
|  |  |  |  |  |  |
